# The Relationship Between Sperm Midpiece Details and DNA Fragmentation in Human Sperm

**DOI:** 10.1007/s43032-025-01887-7

**Published:** 2025-06-02

**Authors:** Muhammed Arif Ibis, Neslihan Oytun Mencik, Burcu Öztürk, Murat Can Karaburun, Cagri Akpinar, Aykut Akıncı, Hakan Bahadır Haberal, Kaan Aydos, Onder Yaman

**Affiliations:** 1 Center for Research On Human Reproduction, Ankara University School of Medicine, Ankara, Turkey; 2 Department of Urology, School of Medicine, Ibn-I Sina Hospital, Ankara University, Altındag/Ankara, Turkey; 3 Department of Urology, Ankara Ataturk Sanatoryum Training and Research Hospital, Ministry of Health, University of Health Sciences, Ankara, Turkey

**Keywords:** DNA fragmentation, Male infertility, Midpiece, Morphometrics, Spermatozoa

## Abstract

Sperm DNA fragmentation is associated with poor sperm quality and reproductive outcomes. Free radicals are a significant cause of DNA fragmentation, with mitochondria being the primary intrinsic source. To investigate the relationship between midpiece measurements containing mitochondria and sperm DNA fragmentation, and to determine the ideal midpiece area. Demographic data, semen analysis results, and DNA fragmentation values were prospectively collected from 239 men with infertility complaints. Detailed analyses and morphometric measurements were performed on 50 spermatozoa from each patient, totaling 11,950 spermatozoa. The ideal DNA fragmentation index (DFI) cutoff value was calculated. Patients were classified into three subgroups based on midpiece length, midpiece width, and midpiece area measurements. The correlation between mid-piece measurements and DFI was investigated. The cutoff value for DFI was determined as 19.50. The odds ratio evaluating the relationship between morphology status and high DFI was 0.159 (95% CI: 0.089—0.282). It was observed that midpiece length and width have statistically significant but low correlations with DFI, whereas midpiece area shows a higher correlation. Finally, based on DFI values, the ideal midpiece area was between 2.31 and 3.13 µm^2^. There is a significant correlation between sperm midpiece area and DFI value, surpassing that of length and width. Future studies may yield important insights by exploring the impact of midpiece area measurements on reproductive outcomes.

## Introduction

Clinical infertility is defined as the inability of a couple to achieve conception following 12 months of unprotected intercourse. Between 20–70% of infertility cases are attributed to male factors, with about 30% of these factors directly affecting fertility [1, 2]. The initial step in evaluating male infertility is a standard semen analysis conducted according to World Health Organization (WHO) guidelines [3]. However, semen analysis has limited diagnostic value in assessing spermatozoa fertilization potential [3–5]. Male fertility can also be influenced by other factors not detected by spermograms, such as genetic and epigenetic factors, and sperm DNA fragmentation [6–8]. Thus, assessing these factors alongside conventional semen analysis may be crucial for evaluating fertility potential.

Intracytoplasmic sperm injection (ICSI) is the only assisted reproductive technique (ART) for patients with cryptozoospermia, severe oligozoospermia, or intrauterine insemination (IUI) failure. On the other hand, recent studies link high sperm DNA fragmentation to poor embryonic development, higher miscarriage rates, and lower implantation success after ART [9, 10]. Eliminating sperm with DNA fragmentation has the potential to improve ICSI outcomes.

While ICSI minimizes the relevance of sperm count, selecting the optimal spermatozoa remains challenging. Conventional swim-up (SU) and discontinuous density gradient centrifugation (DGC) are commonly used sperm preparation methods due to their speed, simplicity, and cost-effectiveness. These methods are intended to eliminate poor-quality sperm based on motility and morphology [11]. However, measuring DNA fragmentation during these processes is nearly impossible. Despite the development of various sperm selection methods, clear improvements in ART success rates have not been achieved [12], likely due to the difficulty in effectively eliminating spermatozoa with high DNA fragmentation.

The midpiece of spermatozoa contains mitochondria that produce energy through oxidative phosphorylation. In light of this information, we investigated the correlation between detailed sperm morphology analysis and midpiece measurements with sperm DNA fragmentation. Given that sperm cells are three-dimensional objects, we included midpiece area calculations in addition to simple length measurements, as length or width alone may not adequately reflect the complexity of their shape.

## Materials and Methods

A prospective study was conducted from December 2022 to June 2024, involving 239 patients presenting with infertility issues at the Ankara University School of Medicine, Center for Research on Human Reproduction. Comprehensive explanations were provided to all participants, and informed consent was obtained prior to their inclusion in the study. Detailed medical histories were collected, and genital physical examinations were performed on all patients. The inclusion criteria were: age between 18 and 45 years, a diagnosis of infertility, and a sperm concentration of at least 1 million/ml in the ejaculate. Patients excluded from the study were those outside the specified age range, those with severe oligozoospermia (sperm concentration below 1 million/ml) or azoospermia, those diagnosed with leukocytospermia, those with a history of genital infection, retrograde ejaculation, genetic disorders, and those undergoing cancer treatment due to malignancies such as a testicular tumor, leukemia, or lymphoma.

In this study, semen analyses and DNA fragmentation index (DFI) values of 239 patients were examined and recorded. Additionally, detailed analyses were performed on a total of 11,950 spermatozoa, with 50 spermatozoa randomly selected from each patient.

In the detailed analysis, defects in the head, neck-midpiece, and tail regions of each spermatozoon were meticulously recorded. Samples were imaged at × 400 magnification using a camera (BRESSER MikrOkular eyepiece camera, Germany) attached to a phase-contrast microscope (Nikon, Japan). Measurements such as head length, head width, midpiece length, midpiece width, and tail length were also taken. Using these measurements, area and ratio calculations were performed. The averages of these values, calculated from 50 spermatozoa for each patient, were recorded as the corresponding data averages for each patient.

Sperm head length was defined as its longest dimension, and head width as the widest region perpendicular to this length. Midpiece length extended from the head base to the tail, with width measured at its widest perpendicular point. The tail length was measured from the midpiece base to the terminal end. The head was considered as an ellipse, and the head area was calculated using the formula"Head area = Head length x Head width x π/4,"with π/4 approximated as 0.79. The midpiece was considered as a rectangle, and the midpiece area was calculated using the formula"Midpiece area = Midpiece length x Midpiece width."

**Fig. 1:**
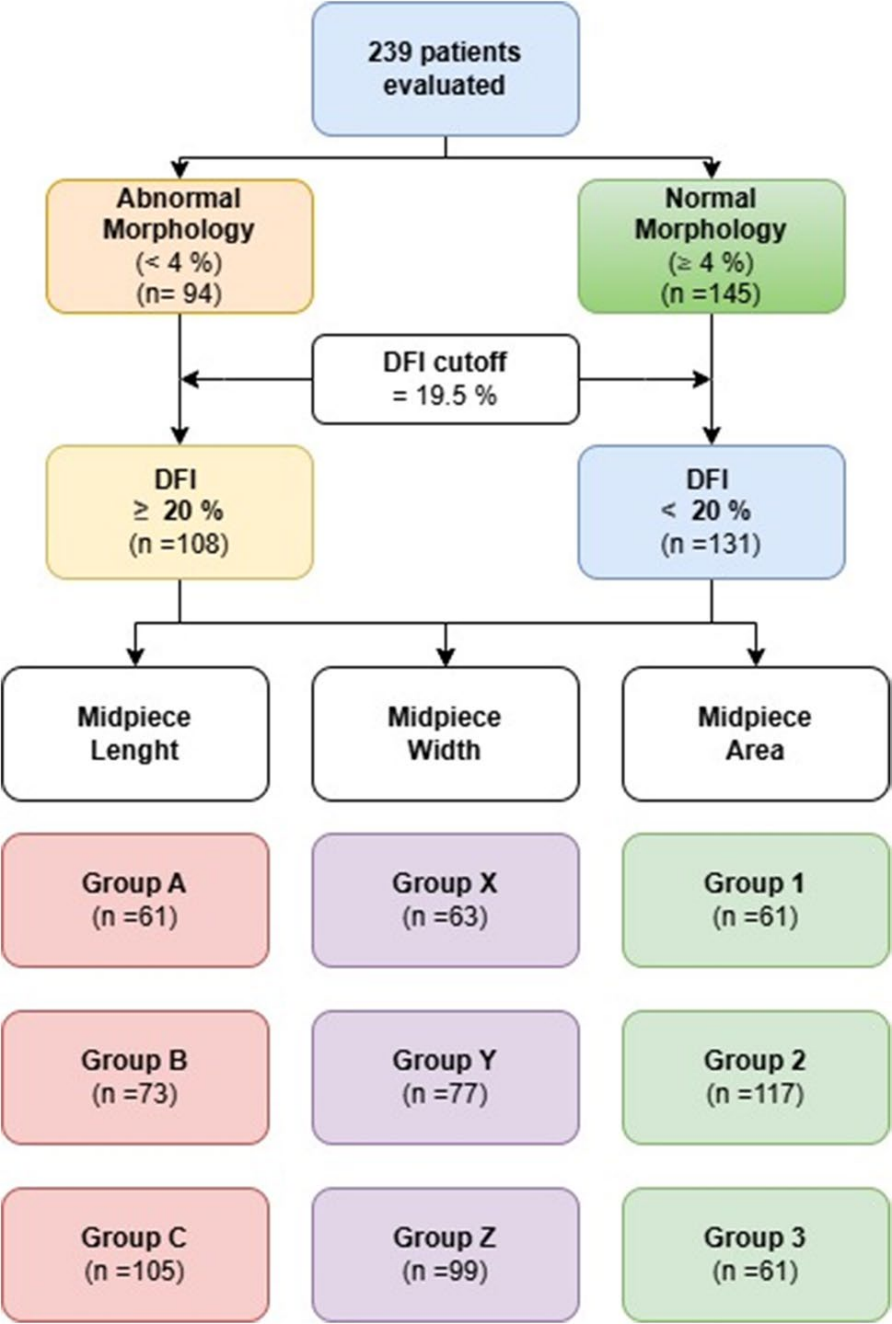
Flowchart illustrating patient stratification based on sperm morphology, DNA fragmentation index (DFI), and midpiece morphometric parameters

The recorded data were compared between groups of patients with abnormal and normal morphology. A Receiver Operating Characteristic (ROC) curve was plotted to differentiate these groups, and a cut-off value of 19.50 was determined at maximum sensitivity and specificity. However, since the patient groups did not differ between 19.50 and 20, and 20 is a more practical whole number, the cut-off value was rounded to 20. Subsequently, patients were divided into two groups based on this cut-off value, and the data were compared between those with DFI values below 20% and those with DFI values at or above 20%. And then, patients were divided into three equal groups based on midpiece length, width, and area measurements. Midpiece length: Group A (3.32–3.9199 µm), Group B (3.92–4.6199 µm), and Group C (4.62–5.22 µm). Midpiece width: Group X (0.45–0.5499 µm), Group Y (0.55–0.6499 µm), and Group Z (0.65–0.75 µm). Midpiece area: Group 1 (1.51–2.3099 µm^2^), Group 2 (2.31–3.1199 µm^2^), and Group 3 (3.12–3.92 µm^2^). The classification of patients based on sperm morphology, DFI, and midpiece dimensions is summarized in Fig. 1.

### Semen Analysis

The basic semen examination was performed in all samples according to the WHO criteria (World Health Organization 2021) [13]. All samples were collected in the clinic through masturbation after a sexual abstinence period of 3 to 5 days. Sperm morphology was evaluated under a 100 × objective, and 200 spermatozoa were classified as having normal or abnormal morphology according to the Tygerberg criteria [14].

### DNA Fragmentation Analysis

Sperm DNA fragmentation was assessed using the TUNEL assay, as described in this study [15], following semen collection and liquefaction. Samples were fixed, permeabilized, embedded in paraffin, and sectioned. After deparaffinization and rehydration, sections were treated with a TUNEL reaction mixture containing TdT enzyme and fluorescein-labeled dUTP to label DNA breaks. Negative and positive controls were included for validation. TUNEL-positive cells, emitting green fluorescence, were quantified under a fluorescence microscope, with DFI calculated as the percentage of TUNEL-positive cells among at least 500 counted cells.

### Statistical Analysis

Statistical analyses were performed using SPSS 25.0.0.1 software (IBM Corp., Armonk, NY, USA). The Shapiro–Wilk test assessed normality. Independent t-test and Mann–Whitney U test compared continuous variables between two groups depending on normality, while categorical variables were analyzed with chi-square tests. For three-group comparisons, ANOVA or Kruskal–Wallis tests were applied depending on normality. ROC analysis identified cut-off values for DFI and sperm midpiece area. Correlations were examined using Pearson or Spearman tests. Significant three-group results underwent Bonferroni-corrected pairwise comparisons (adjusted *p* < 0.017). Statistical significance was set at *p* < 0.05.

## Results

**Fig. 2:**
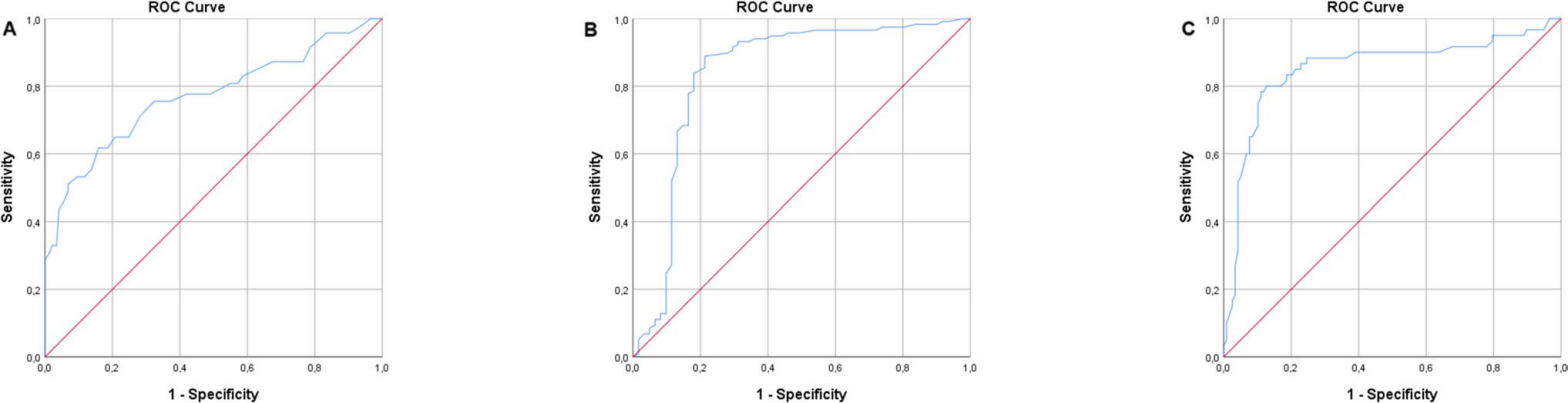
ROC curve analysis: **A**) ROC curve analysis of DNA Fragmentation Index (DFI) levels in patients with normal and abnormal sperm morphology. **B**) ROC curve analysis of the midpiece area in patients from the combined Group 1 and Group 2. **C**) ROC curve analysis of the midpiece area in patients from the combined Group 2 and Group 3

This study included 239 patients aged between 19 and 43 years, with a mean age of 29.84 ± 4.85 years. Patients were compared based on abnormal morphology (< 4%) and normal morphology (≥ 4%), as shown in Table 1. The abnormal morphology group included 94 patients, while the normal morphology group included 145 patients. The sperm concentration and motility were significantly lower in the abnormal morphology group compared to the normal morphology group (*p* = 0.002 and *p* < 0.001, respectively). Detailed morphology analysis showed that the proportion of spermatozoa in the"Normal"category across the head, midpiece, and tail was significantly higher in the normal morphology group, while the thin-thick defect across the middle piece defects was less. Another significant finding was the difference in DFI between the abnormal morphology group and the normal morphology group (*p* < 0.001). Based on patients categorized by abnormal morphology and normal morphology, a ROC curve was plotted for DFI levels (Fig. 2A). The cutoff value for DFI, providing maximum sensitivity (71.3%) and specificity (71.7%), was determined as 19.50, with an area under the curve (AUC) value calculated as 0.769.

**Table 1:** Basic characteristics, semen analysis, and detailed sperm morphology for all patients and by morphology

	All patients	Abnormal Morphology (< %4) (*n* = 94)	Normal Morphology (≥ %4) (*n* = 145)	*p* value
Age, year	29.84 ± 4.85	30.47 ± 5.43	29.43 ± 4.41	0.105
Body mass index, (kg/m^2^)	29.02 ± 4.96	28.89 ± 5.06	29.10 ± 4.91	0.748
Varicocele				0.011
Absent, *n* (%)	169 (100)	56 (33.1)	113 (66.9)	
Grade 1, *n* (%)	22 (100)	10 (45.5)	12 (54.5)	
Grade 2, *n* (%)	35 (100)	19 (54.3)	16 (45.7)	
Grade 3, *n* (%)	13 (100)	9 (69.2)	4 (30.8)	
Smoking, *n* (%)		28 (29.8)	28 (19.3)	0.062
Abstinence Duration, day	3.27 ± 0.61	3.30 ± 0.62	3.26 ± 0.60	0.473
Semen Volume, (mL)	2.89 ± 0.76	2.96 ± 0.73	2.84 ± 0.77	0.247
Concentration, (× 10^6^/mL)	15.80 ± 10.01	13.37 ± 9.63	17.37 ± 9.98	0.002
Motility, (%)	49.23 ± 18.93	38.53 ± 18.43	56.17 ± 15.80	< 0.001
Kruger, (%)				
Morphologically Normal Spermatozoa	3.89 ± 2.49	1.10 ± 1.21	5.70 ± 0.95	< 0.001
Head Defects	69.22 ± 8.52	70.91 ± 11.76	68.12 ± 5.24	0.154
Neck and Midpiece Defects	19.91 ± 7.30	20.87 ± 10.21	19.28 ± 4.44	0.097
Tail Defects	6.99 ± 6.16	7.12 ± 6.79	6.90 ± 5.74	0.773
Excessive Residual Cytoplasm	1.42 ± 1.03	2.18 ± 1.20	0.93 ± 0.47	< 0.001
Head Defects				
Normal	11.51 ± 5.31	10.24 ± 6.41	12.32 ± 4.28	0.023
Amorphous	26.00 ± 5.55	26.71 ± 7.16	25.53 ± 4.15	0.611
Pyriform	4.64 ± 2.74	4.93 ± 3.04	4.46 ± 2.51	0.542
Conical	4.49 ± 2.88	4.61 ± 2.99	4.42 ± 2.82	0.750
Round	3.36 ± 2.60	3.51 ± 3.15	3.26 ± 2.18	0.491
Neck and Midpiece Defects				
Normal	35.13 ± 3.51	34.48 ± 3.38	35.54 ± 3.54	0.010
Asymmetric	3.00 ± 1.80	2.84 ± 1.79	3.11 ± 1.80	0.178
Thin-Thick	5.98 ± 2.34	6.64 ± 2.32	5.56 ± 2.27	0.003
Broken	3.16 ± 1.80	3.14 ± 1.81	3.18 ± 1.80	0.676
Irregular	2.72 ± 1.93	2.90 ± 1.76	2.61 ± 1.87	0.159
Tail Defects				
Normal	39.83 ± 3.70	39.38 ± 3.16	40.12 ± 4.00	0.025
Broken	2.46 ± 1.74	2.63 ± 1.86	2.36 ± 1.66	0.297
Curly	1.95 ± 1.56	2.06 ± 1.64	1.87 ± 1.50	0.220
Short	2.00 ± 1.62	2.12 ± 1.65	1.92 ± 1.61	0.288
Irregular	2.76 ± 1.93	2.84 ± 1.93	2.70 ± 1.94	0.568
Double	0.33 ± 0.53	0.34 ± 0.61	0.32 ± 0.47	0.578
Other	0.67 ± 0.67	0.63 ± 0.64	0.70 ± 0.69	0.460
Head Length, (µm)	4.68 ± 0.70	4.76 ± 0.48	4.62 ± 0.81	0.148
Head Width, (µm)	2.80 ± 4.17	2.74 ± 0.39	2.84 ± 0.43	0.074
Head Area, (µm^2^)	10.37 ± 2.28	10.40 ± 2.22	10.35 ± 2.32	0.868
Midpiece Length, (µm)	4.40 ± 0.58	4.36 ± 0.66	4.42 ± 0.52	0.387
Midpiece Width, (µm)	0.61 ± 0.09	0.61 ± 0.11	0.62 ± 0.08	0.710
Midpiece Area, (µm^2^)	2.72 ± 0.62	2.71 ± 0.83	2.72 ± 0.43	0.981
Tail Length, (µm)	50.47 ± 8.43	50.08 ± 6.04	50.72 ± 9.68	0.565
Ratio of Midpiece Length to Head Length	0.96 ± 0.20	0.93 ± 0.17	0.99 ± 0.22	0.019
Ratio of Midpiece Length to Tail Length	0.09 ± 0.02	0.09 ± 0.02	0.09 ± 0.02	0.376
Ratio of Tail Length to Head Length	10.85 ± 1.50	11.05 ± 1.77	10.54 ± 0.84	0.009
Ratio of Midpiece Area to Head Area	0.28 ± 0.10	0.28 ± 0.12	0.28 ± 0.08	0.922
DNA Fragmentation Index (%)	19.93 ± 12.60	27.65 ± 14.02	14.92 ± 8.46	< 0.001

There were 131 patients in the DFI < 20% group and 108 patients in the DFI ≥ 20% group (Table 2). The presence of varicocele was significantly higher in the DFI ≥ 20% group (*p* = 0.024), with a higher frequency of Grade 2 and Grade 3 varicoceles in this group. Additionally, the rate of smoking was significantly higher in the DFI ≥ 20% group (*p* = 0.018), with approximately one-third of this group being smokers. Sperm concentration, motility, and morphology were higher in the DFI < 20% group (*p* = 0.027, *p* = 0.001, and *p* < 0.001, respectively). Among patients with abnormal morphology, 71.3% had a DFI ≥ 20%, whereas among those with normal morphology, this rate was 28.3%. This difference was statistically significant (*p* < 0.001). The odds ratio evaluating the relationship between morphology and high DFI was 0.159 (95% CI: 0.089—0.282). The rates of neck and midpiece defects and excessive residual cytoplasm were higher in the DFI ≥ 20% group than in the DFI < 20% group (*p* = 0.013 and *p* = 0.005, respectively). The thin-thick defect across the middle piece defects was less in the DFI < 20% group (*p* = 0.045).

**Table 2:** Basic characteristics, semen analysis, and detailed sperm morphology between DNA fragmentation index (DFI) < 20% and DFI ≥ 20%

	DFI < %20 (*n* = 131)	DFI ≥ %20 (*n* = 108)	*p* value
Age, year	29.69 ± 4.73	30.02 ± 5.01	0.600
Body mass index, (kg/m^2^)	28.47 ± 4.93	29.68 ± 4.94	0.061
Varicocele			0.024
Absent, *n* (%)	103 (60.9)	66 (39.1)	
Grade 1, *n* (%)	10 (45.5)	12 (54.5)	
Grade 2, *n* (%)	14 (40)	21 (60)	
Grade 3, *n* (%)	4 (30.8)	9 (69.2)	
Smoking, *n* (%)	24 (18.3)	34 (31.5)	0.018
Abstinence Duration, day	3.24 ± 0.55	3.32 ± 0.67	0.330
Semen volume, (mL)	2.87 ± 0.81	2.91 ± 0.69	0.706
Concentration, (× 10^6^/mL)	17.10 ± 10.29	14.22 ± 9.49	0.027
Motility, (%)	53.00 ± 16.53	44.66 ± 20.65	0.001
Morphology			< 0.001
Anormal morfoloji (< %4), n (%)	27 (28.7)	67 (71.3)	
Normal morfoloji (≥ %4)	104 (71.7)	41 (28.3)	
Kruger, (%)			
Morphologically Normal Spermatozoa	4.98 ± 1.74	2.56 ± 2.62	< 0.001
Head Defects	69.38 ± 7.34	69.02 ± 9.79	0.716
Neck and Midpiece Defects	18.73 ± 6.23	21.34 ± 8.23	0.013
Tail Defects	6.91 ± 6.88	7.08 ± 5.19	0.189
Excessive Residual Cytoplasm	1.24 ± 0.88	1.64 ± 1.16	0.005
Head Defects			
Normal	12.34 ± 4.73	10.50 ± 5.79	0.047
Amorphous	25.99 ± 4.81	26.00 ± 6.35	0.444
Pyriform	4.44 ± 2.64	4.90 ± 2.84	0.292
Conical	4.18 ± 2.70	4.88 ± 3.06	0.082
Round	3.06 ± 2.27	3.72 ± 2.93	0.127
Neck and Midpiece Defects			
Normal	35.60 ± 3.51	34.56 ± 3.44	0.030
Asymmetric	3.07 ± 1.67	2.93 ± 1.94	0.371
Thin-Thick	5.71 ± 2.25	6.31 ± 2.43	0.045
Broken	3.01 ± 1.74	3.35 ± 1.86	0.120
Irregular	2.62 ± 1.94	2.85 ± 1.70	0.181
Tail Defects			
Normal	40.26 ± 3.77	39.31 ± 3.57	0.040
Broken	2.44 ± 1.73	2.50 ± 1.76	0.787
Curly	1.88 ± 1.40	2.03 ± 1.73	0.763
Short	1.88 ± 1.55	2.14 ± 1.71	0.259
Irregular	2.53 ± 1.84	3.03 ± 2.02	0.067
Double	0.34 ± 0.49	0.31 ± 0.57	0.319
Other	0.67 ± 0.70	0.68 ± 0.64	0.786
Head Length, (µm)	4.64 ± 0.76	4.72 ± 0.63	0.401
Head Width, (µm)	2.79 ± 0.44	2.82 ± 0.38	0.573
Head Area, (µm^2^)	10.21 ± 2.31	10.55 ± 2.23	0.252
Midpiece Length, (µm)	4.44 ± 0.49	4.34 ± 0.67	0.183
Midpiece Width, (µm)	0.62 ± 0.08	0.61 ± 0.11	0.526
Midpiece Area, (µm^2^)	2.73 ± 0.40	2.70 ± 0.81	0.691
Tail Length, (µm)	50.45 ± 9.17	50.49 ± 7.47	0.969
Ratio of Midpiece Length to Head Length	0.99 ± 0.21	0.94 ± 0.19	0.056
Ratio of Midpiece Length to Tail Length	0.09 ± 0.02	0.09 ± 0.02	0.184
Ratio of Tail Length to Head Length	10.97 ± 1.89	10.71 ± 0.79	0.156
Ratio of Midpiece Area to Head Area	0.28 ± 0.09	0.27 ± 0.10	0.203
DNA Fragmentation Index (%)	10.73 ± 5.36	31.07 ± 9.48	< 0.001

**Fig. 3:**
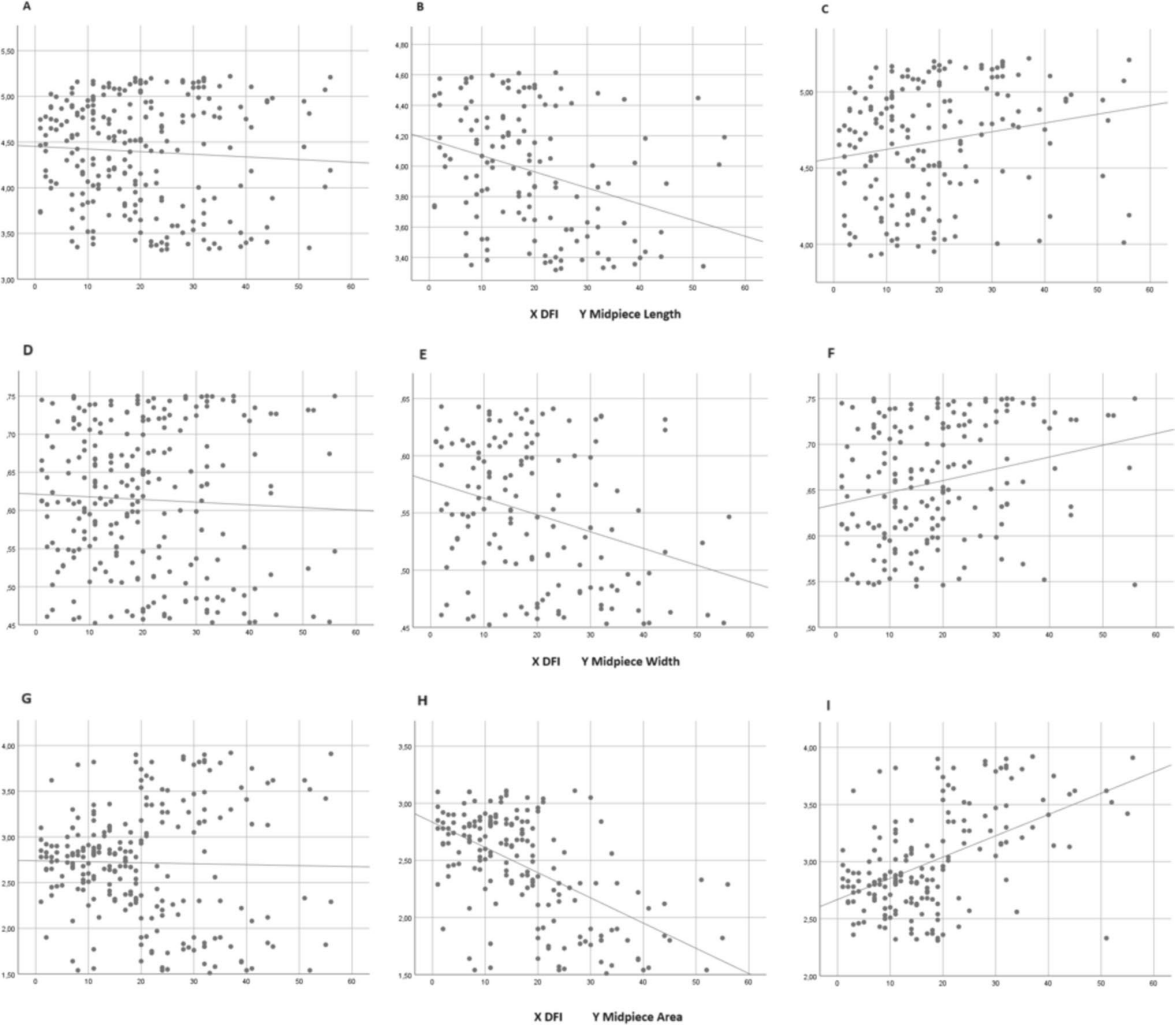
Correlation between DNA Fragmentation Index (DFI) and midpiece length, width, and area. For midpiece length: **A**) all patients, **B**) patients from the combined Group A and Group B, and **C**) patients from the combined Group B and Group C. For Midpiece Width: **D**) all patients, **E**) patients from the combined Group X and Group Y, and **F**) patients from the combined Group Y and Group Z. For Midpiece Area: **G**) all patients, H) patients from the combined Group 1 and Group 2, and **I**) patients from the combined Group 2 and Group 3

No significant correlations were found between DFI and midpiece length, width, or area (*p* = 0.422, *p* = 0.232, and *p* = 0.742, respectively) (Table 3). Figures 3A, D, and G display the corresponding correlation graphs.

**Table 3:** Correlation results between midpiece measurements and DNA Fragmentation Index (DFI) in all patients and subgroups

	Correlation coefficient (r)	p Value (p)
Midpiece Length		
All patients	0.052	0.422
Group A ve Group B Patients	−0.320	< 0.001
Group B ve Group C Patients	0.195	0.009
Midpiece Width		
All patients	0.078	0.232
Group X ve Group Y Patients	−0.279	0.001
Group Y ve Group Z Patients	0.246	0.001
Midpiece Area		
All patients	0.021	0.742
Group 1 ve Group 2 Patients	−0.529	< 0.001
Group 2 ve Group 3 Patients	0.460	< 0.001

Patients were divided into three equal groups based on midpiece length, width, and area measurements (Table 4). A significant difference in sperm concentration was observed between Group A and Group C (*p* = 0.003) among midpiece length subgroups. Group A and Group B also differed significantly in excessive residual cytoplasm (*p* = 0.011). For DFI values, Group A and Group B showed a significant difference (*p* < 0.001).

**Table 4:** Comparison of semen analysis and DNA Fragmentation Index (DFI) values among patients grouped by midpiece length, midpiece width, midpiece area

	Midpiece Length							Midpiece Width							Midpiece Area						
	Group A (*n* = 61)	Gorup B (*n* = 73)	Group C (*n* = 105)	*p* Value (*p*)	Group A vs. Group B	Group A vs. Group C	Group B vs. Group C	Group X (*n* = 63)	Group Y (*n* = 77)	Group Z (*n* = 99)	*p* Değeri (*p*)	Group X vs. Group Y	Group X vs. Group Z	Group Y vs. Group Z	Group 1 (*n* = 61)	Group 2 (*n* = 117)	Group 3 (*n* = 61)	*p* Value (*p*)	Group 1 vs. Group 2	Group 1 vs. Group 3	Group 2 vs. Group 3
Semen Volume, (mL)	2.90 ± 0.79	2.78 ± 0.71	2.95 ± 0.76	0.427				2.78 ± 0.73	2.92 ± 0.72	2.93 ± 0.80	0.382				2.82 ± 0.67	2.85 ± 0.81	3.02 ± 0.72	0.313			
Concentration, (× 10^6^/mL)	12.61 ± 8.22	16.12 ± 9.68	17.44 ± 10.80	0.013	0.049	**0.003**	0.557	13.95 ± 9.93	16.73 ± 10.23	16.26 ± 9.83	0.139				12.10 ± 9.02	17.67 ± 9.22	15.92 ± 11.46	< 0.001	** < 0.001**	0.023	0.050
Motility, (%)	47.72 ± 20.21	51.56 ± 16.57	48.49 ± 19.71	0.528				48.67 ± 19.50	53.17 ± 18.72	46.53 ± 18.39	0.060				48.56 ± 20.69	52.89 ± 16.62	42.89 ± 19.80	0.013	0.303	0.133	**0.003**
Kruger, (%)																					
Morphologically Normal Spermatozoa	3.36 ± 2.57	4.53 ± 2.17	3.74 ± 2.57	0.050				3.33 ± 2.60	4.75 ± 2.21	3.57 ± 2.47	0.002	**0.003**	0.679	**0.001**	2.67 ± 2.49	5.32 ± 1.62	2.36 ± 2.34	< 0.001	** < 0.001**	0.486	** < 0.001**
Head Defects	69.92 ± 9.45	69.10 ± 7.48	68.90 ± 8.69	0.648				69.97 ± 10.26	68.00 ± 6.73	69.69 ± 8.53	0.573				70.02 ± 10.63	68.23 ± 6.04	70.31 ± 10.03	0.487			
Neck and Midpiece Defects	19.26 ± 7.61	20.00 ± 6.83	20.22 ± 7.49	0.536				18.71 ± 8.05	20.29 ± 5.90	20.37 ± 7.76	0.282				19.66 ± 8.82	19.68 ± 5.17	20.59 ± 9.03	0.645			
Tail Defects	7.46 ± 6.60	6.37 ± 6.48	7.14 ± 5.68	0.214				7.98 ± 6.75	6.96 ± 6.25	6.37 ± 5.67	0.310				7.66 ± 7.26	6.77 ± 5.89	6.74 ± 5.50	0.731			
Excessive Residual Cytoplasm	1.69 ± 1.78	1.19 ± 0.89	1.43 ± 1.01	0.035	**0.011**	0.201	0.098	1.76 ± 1.12	1.14 ± 0.93	1.42 ± 1.00	0.002	** < 0.001**	0.068	0.031	1.87 ± 1.19	1.05 ± 0.67	1.69 ± 1.19	< 0.001	** < 0.001**	0.474	** < 0.001**
DNA Fragmentation Index, (%)	23.56 ± 11.63	16.42 ± 11.68	20.27 ± 13.20	0.001	** < 0.001**	0.055	0.053	23.89 ± 13.12	16.01 ± 10.92	20.46 ± 12.71	< 0.001	** < 0.001**	0.078	**0.013**	27.03 ± 12.19	12.40 ± 7.75	27.25 ± 12.24	< 0.001	** < 0.001**	0.984	** < 0.001**

When comparing midpiece width subgroups, Group Y had significantly better results in the percentage of morphologically normal spermatozoa and DFI values than Groups X and Z. Additionally, significant differences in excessive residual cytoplasm were observed between Group X and Group Y (*p* < 0.001).

In midpiece area subgroups, Group 2 showed higher sperm concentration than Group 1 and higher sperm motility than Group 3. Group 2 also had superior results in normal sperm morphology, excessive residual cytoplasm, and DFI compared to Groups 1 and 3.

Table 3 shows the correlation coefficients and significance levels between DFI and midpiece dimensions in subgroups. For midpiece length, significant correlations were found: between Group A and Group B (r = −0.320, *p* < 0.001), indicating a weak to moderate negative relationship, and between Group B and Group C (r = 0.195, *p* = 0.009), indicating a weak positive relationship (Figs. 3B and C). For midpiece width, significant correlations were observed: between Group X and Group Y (r = −0.279, *p* = 0.001), indicating a weak negative relationship, and between Group Y and Group Z (r = 0.246, *p* = 0.001), indicating a weak positive relationship (Figs. 3E and F). For midpiece area, a moderate negative correlation was found between Group 1 and Group 2 (r = −0.529, *p* < 0.001), while a moderate positive correlation was found between Group 2 and Group 3 (r = 0.460, *p* < 0.001) (Figs. 3H and I). In summary, midpiece area demonstrated a stronger correlation with DFI compared to length and width.

After identifying the strongest correlation with the midpiece area, we determined ideal midpiece area cutoff values. The lower cutoff, calculated from Groups 1 and 2, was set at 2.31 µm^2^, providing maximum sensitivity (89%) and specificity (78.7%) for DFI (AUC: 0.838) (Fig. 2B). The upper cutoff, derived from Groups 2 and 3, was 3.13 µm^2^, offering maximum sensitivity (78.3%) and specificity (89%) for DFI (AUC: 0.853) (Fig. 2C). These results indicated that the ideal midpiece area should be between 2.31 and 3.13 µm^2^.

In a subgroup of 43 normozoospermic men whose partners were diagnosed with a female infertility factor, 28 individuals (65.1%) exhibited a DFI < 20%. In the overall cohort, the proportion of patients with DFI < 20% was 54.8% (131/239). Among these 43 men, 27 (62.8%) had a sperm midpiece area within the range of 2.31–3.13 µm^2^. Notably, this morphometric characteristic was present in 85.7% of cases with DFI < 20% (24/28), whereas it was observed in only 20% (3/15) of those with DFI > 20%, indicating a statistically significant difference (*p* < 0.001).

## Dıscussion

This study indicated a cutoff value of 19.50 for DFI, with teratozoospermic men having approximately 6 times higher likelihood of DFI ≥ 20 compared to men with normal morphology (1/0.159). The data highlighted that DFI correlates significantly not only with standard semen analysis but also with detailed sperm morphological analysis. Specifically, among these morphological defects, the"thin-thick"defect in the midpiece was significantly more prevalent in the group with DFI ≥ 20. Importantly, when analyzing sperm midpiece measurements, it was observed that midpiece length and width have statistically significant but low correlations with DFI, whereas midpiece area shows a higher correlation. Finally, based on DFI values, the ideal midpiece area was provided for the first time in a study.

As is well known, mitochondria are located in the midpiece of sperm and serve as a source of free radicals (FRs). FRs play dual roles in physiological processes [16]. Low levels of reactive oxygen species are necessary for spermatozoa functions like capacitation, acrosome reaction, and fertilization, but high levels can damage critical biomolecules, including nucleic acids and protamines essential for spermatozoa stability. Superoxide, a byproduct of mitochondrial metabolism, is generated at complexes I and III of the electron transport chain. It is then converted to H2O2 by mitochondrial superoxide dismutase and then detoxified by catalase and glutathione peroxidase. Imbalances in production or detoxification can increase free radical levels, which lead to various alterations in nuclear DNA, including single and double-strand breaks. MacLeod’s 1943 finding that human sperm produces hydrogen peroxide initiated a line of research that led to modern techniques for measuring oxidative stress in the semen of infertile men [17].

Both a decrease and an increase in midpiece area appear to be negatively associated with DNA integrity, as reflected by elevated DFI levels. Maintaining midpiece dimensions within an optimal range may be more critical than simply increasing or decreasing their size. Various intrinsic and extrinsic factors, including oxidative stress, environmental toxins, lifestyle factors, and age, may contribute to alterations in midpiece dimensions by promoting DNA fragmentation [18]. In particular, mitochondrial dysfunction—often triggered by elevated levels of reactive oxygen species [19]—may impair midpiece development. To preserve DNA integrity and thereby optimize midpiece dimensions, potential strategies may include antioxidant therapy, dietary modifications, and hormonal regulation [20].

Research on sperm midpiece measurements dates back to the late 1990 s and includes studies on both animal and human spermatozoa. Anderson MJ et al. [21] examined sperm morphology in 123 species with varying mating systems and found that species with polyandrous females exhibited a significant increase in midpiece volume. This suggests that increased mitochondrial content, associated with enhanced sperm motility, may be an evolutionary adaptation driven by sperm competition, meriting further investigation.

Thirteen years after Anderson MJ et al.'s study, another research focused on the midpiece measurements of sperm in the bird species *Taeniopygia guttata* [22]. The study showed that mitochondrial density does not vary with midpiece length, and spermatozoa with longer midpieces were proportionally thinner, despite having similar crista density. These findings suggested that structural complexity in sperm midpieces might have been better described by alternative measurements such as area rather than linear dimensions alone. Another study [23] on the same bird species found a positive correlation between sperm length and swimming speed, which decreased in the longest sperm. The fastest spermatozoa had a similar midpiece, regardless of total spermatozoa length. Additionally, sperm with shorter midpieces have been shown to contain higher concentrations of stored intracellular ATP. These results suggest the need to find optimal value ranges for alternative measurements, such as area, rather than relying solely on linear dimensions for the midpiece.

Recent advancements have moved research on sperm midpiece measurements to a new level and a study was published investigating whether sperm midpiece measurements change during physiological processes [24]. The study found that capacitation reduced mitochondrial area, mainly by decreasing midpiece width rather than length. Following the acrosome reaction, both midpiece area and width further decreased, with minimal change in length. While capacitation is known to induce morphological and physiological changes in spermatozoa for fertilization, this study also clearly revealed changes in mitochondrial and midpiece dimensions.

The animal studies mentioned above have highlighted the complex structure of the sperm midpiece, the need for developing measurements across different dimensions, and changes in size during physiological processes. However, what do studies on human spermatozoa midpiece measurements reveal? In 2013, Mossman JA and colleagues reported that tail length and total sperm length were positively correlated with total sperm count, sperm concentration, and total motile sperm count, while midpiece length was only correlated with total motile sperm count [25]. An interesting finding from this study was that the lack of correlation between average midpiece length and other length measurements suggested an allometric relationship between midpiece formation and the synthesis of other components. Our study similarly revealed a statistical difference in the midpiece-to-head length ratio between abnormal and normal morphology patient groups. This may indicate that normal morphology spermatozoa exhibit more pronounced allometry compared to abnormal morphology spermatozoa.

Contrary to our findings, Nguyen HTT et al. [26] did not find significant differences in semen parameters or detailed sperm morphology between normal and high DFI groups. This discrepancy may stem from uncertainties in the DFI cutoff value. Nguyen HTT et al. used a 15% DFI cutoff based on prior studies, but no standard DFI cutoff currently exists. Therefore, until a standard is established, researchers should determine their own DFI cutoff values using statistical methods on their data, as varying cutoffs can influence results. Another study [27] established an 18% DFI cutoff through ROC analysis and showed that patients with high DFI were 4.6 times more likely to have abnormal semen morphology. While correlations between DFI and various semen parameters and morphological defects were found, this study did not evaluate the dimensions of sperm subcomponents.

Despite extensive research on both animal and human spermatozoa, the complexities of sperm midpiece morphology remain incompletely understood, underscoring the need for broader studies. The changes in midpiece areas throughout the spermatozoa lifecycle are intriguing. Since sperm are three-dimensional, simple length measurements may not fully capture their structural complexity. Therefore, incorporating non-linear measurements, such as the area or volume of sperm subcomponents, may provide more detailed insights into sperm morphology. In our study, we could identify the sperm midpiece area as a significant parameter.

Conventional sperm preparation methods such as SU and DGC have long served as standard techniques in assisted reproduction due to their simplicity, speed, and cost-effectiveness [11]. However, their efficiency decreases notably in cases of severe oligoasthenoteratozoospermia, prompting the development of more refined sperm selection strategies [28]. Recent advancements have introduced Advanced Sperm Selection Techniques, which aim to improve the selection of spermatozoa with superior functional and genetic characteristics. These include Microfluidic Sperm Sorting, Zeta Potential Selection, Magnetic-Activated Cell Sorting (MACS), Laser-Assisted Immobile Sperm Selection (LAISS), and Intracytoplasmic Morphologically Selected Sperm Injection (IMSI). Each method utilizes distinct principles—such as fluid dynamics, membrane charge, magnetic labeling, laser targeting, or high-magnification microscopy—to isolate sperm with higher fertilization potential.

These emerging technologies aim to isolate the highest-quality spermatozoa based on distinct physiological traits, offering a promising future for more effective and personalized fertility treatments through their potential synergistic use. While advanced sperm selection techniques aim to isolate spermatozoa of the highest quality, the ultimate goal in assisted reproduction remains the improvement of live birth rates. Sperm DNA fragmentation is a key factor affecting pregnancy outcomes, and studies have demonstrated that various selection methods can enrich for sperm with better DNA integrity [29]. Nonetheless, approaches like MACS, LAISS, and microfluidics, despite their potential, involve higher costs and longer processing times, which limit their practicality in routine use. Although no single technique has been established as the definitive gold standard, current evidence indicates that these newer methods offer improvements over traditional SU and DGC techniques [28].

Thus, there remains a need for simpler and more specific techniques to identify high-quality sperm. Among advanced techniques, IMSI stands out for its meticulous evaluation of sperm morphology using high-magnification microscopy. Although convincing clinical evidence still remains limited, the meticulous selection process of IMSI has been reported to reduce the transmission of genetic abnormalities or DNA damage to the embryo, potentially may leading to improved pregnancy outcomes [30]. However, the equipment requirements and the need for specialized training contribute to higher costs and procedural complexity, potentially limiting its accessibility. Exploring cost-effective equipment alternatives could enhance the accessibility of IMSI in clinical settings. In this context, although the technique we employed may not be sufficient on its own to identify the most suitable sperm for ICSI or IVF, it can serve as a practical and readily available adjunct to support embryologists in optimizing the sperm selection process.

This study has several limitations. Firstly, while electron microscopy was not employed, the light microscopy and magnifications employed were sufficient to ensure accurate results. We are planning to initiate a follow-up study utilizing electron microscopy to achieve enhanced morphological detail. Secondly, while sperm DNA damage values may not provide definitive insights into the DNA quality of selected sperm during ICSI, they offer a general opinion. Lastly, the study does not include reproductive outcomes; however, our future analyses will integrate data from ongoing and new cases to evaluate the impact of midpiece measurements on reproductive results.

## Conclusion

This study is the first to compare sperm midpiece measurements with DNA fragmentation and propose an optimal midpiece area of 2.31–3.13 µm^2^, with deviations linked to higher fragmentation rates. Including midpiece measurements alongside motility and morphology in sperm selection may improve ICSI outcomes. Further research is needed to clarify the role of midpiece structure in fertility.

## Data Availability

Data available on request from the authors.
